# Prediction of Tacrolimus Exposure by *CYP3A5* Genotype and Exposure of Co-Administered Everolimus in Japanese Renal Transplant Recipients

**DOI:** 10.3390/ijms19030882

**Published:** 2018-03-16

**Authors:** Hideaki Kagaya, Takenori Niioka, Mitsuru Saito, Takamitsu Inoue, Kazuyuki Numakura, Ryohei Yamamoto, Yumiko Akamine, Tomonori Habuchi, Shigeru Satoh, Masatomo Miura

**Affiliations:** 1 Department of Pharmacy, Akita University Hospital, 1-1-1 Hondo, Akita 010-8543, Japan; hideaki-kagaya@hos.akita-u.ac.jp (H.K.); t-niioka@hirosaki-u.ac.jp (T.N.); yumiko-ai@hos.akita-u.ac.jp (Y.A.); 2 Department of Urology, Akita University School of Medicine, 1-1-1 Hondo, Akita 010-8543, Japan; mitsaito@med.akita-u.ac.jp (M.S.); takamitu@doc.med.akita-u.ac.jp (T.I.); numakura@doc.med.akita-u.ac.jp (K.N.); yama815@med.akita-u.ac.jp (R.Y.); thabuchi@doc.med.akita-u.ac.jp (T.H.); 3 Center for Kidney Disease and Transplantation, Akita University Hospital, 1-1-1 Hondo, Akita 010-8543, Japan; shigerus@doc.med.akita-u.ac.jp

**Keywords:** tacrolimus, everolimus, *CYP3A5* polymorphism, renal transplantation

## Abstract

While tacrolimus and everolimus have common metabolic pathways through CYP3A4/5, tacrolimus is metabolized solely by CYP3A4 in recipients with the CYP3A5*3/*3. The purpose of this study was to evaluate how the area under the blood concentration-time curves (AUC) of tacrolimus could be predicted based on *CYP3A5* genotype and the AUC of everolimus in renal transplant patients taking both drugs. The dose-adjusted AUC (AUC/D) of tacrolimus and everolimus were calculated at one month and one year after transplantation. Significant correlations between the AUC/D of tacrolimus and everolimus were found for patients with the CYP3A5*1 allele or CYP3A5*3/*3 at both one month and one year. At both stages, the determination coefficients were higher and the slopes of regression equations were larger for patients with CYP3A5*3/*3 compared to the CYP3A5*1 allele. A good correlation between single doses of tacrolimus and everolimus was found for CYP3A5*3/*3 patients at 1 year after transplantation (*r* = 0.794, *p* < 0.001). The variability of the AUC_0–24_/D of tacrolimus for each *CYP3A5* genotype could be predicted based on the AUC_0–12_/D of everolimus. Clinicians may be able to comprehensively carry out the dose adjustments of tacrolimus and everolimus based on relationship with AUCs of both drugs in each *CYP3A5* genotype.

## 1. Introduction

Tacrolimus, one of the calcineurin inhibitors (CNIs), displays high intra- and inter-individual pharmacokinetic variability and a poor correlation between dosage and drug blood concentration [1,2]. In addition, the therapeutic window of tacrolimus blood concentration is very narrow [1,2]. Hence, most clinicians prescribing tacrolimus use therapeutic drug monitoring (TDM) to guide dosing. Tacrolimus is mainly metabolized by cytochrome P450 (CYP) 3A4/5, which is expressed in the small intestine and hepatocytes, and the variability in tacrolimus pharmacokinetics has been attributed to individual differences in expression of the CYP3A4/5 protein [1,2]. The expression of *CYP3A5* protein in the liver and small intestine is strongly correlated with a single nucleotide polymorphism, 6986A > G, within intron 3 of *CYP3A5*, designated CYP3A5*3 [3]. Tacrolimus pharmacokinetics is affected by the *CYP3A5* polymorphism, as maintaining the same target blood concentration in recipients with CYP3A5*3/*3 requires a significantly lower dose of tacrolimus than in those with the CYP3A5*1 allele [4].

Recently, the addition of everolimus, a rapamycin derivative inhibitor of mTORi, into immunosuppressive therapy including tacrolimus has been used to reduce the risk of tacrolimus-induced nephrotoxicity in renal transplant recipients [5,6,7,8,9]. Everolimus also exhibits a narrow therapeutic window of blood concentration similar to tacrolimus [10]. Therefore, individualized dosage based on blood concentration is very important for renal transplant recipients taking tacrolimus and everolimus. Although everolimus is primarily metabolized by CYP3A4 and *CYP3A5* [10], its pharmacokinetics are reported to be unaffected by *CYP3A5* polymorphisms [11,12,13]. Thus, CYP3A4 rather than *CYP3A5* is most likely the predominant enzyme involved in metabolic clearance of everolimus, whereas tacrolimus is metabolized by *CYP3A5* rather than CYP3A4.

Tacrolimus [1,2] and everolimus [10] share a common metabolic pathway through CYP3A4/5; however, in patients with CYP3A5*3/*3, tacrolimus is metabolized solely by CYP3A4. The purpose of this study was to evaluate how the area under the blood concentration-time curve (AUC) of tacrolimus could be predicted based on *CYP3A5* genotype and the AUCs of everolimus in renal transplant recipients taking both drugs.

## 2. Results

Clinical characteristics of the renal transplant recipients are listed in Table 1. The allele frequencies for CYP3A5*1 and *3 at 1 month after renal transplantation (*n* = 50) were 30.0% and 70.0%, respectively. Thirty-one of the 50 patients remained in the study 1 year after transplantation. The allele frequencies for CYP3A5*1 and *3 at 1 year after renal transplantation (*n* = 31) were 25.0% and 75.0%, respectively. The allele frequency of CYP3A5*3 was in Hardy–Weinberg equilibrium [14]. There were significant differences in clinical characteristics such as body weight, aspartate aminotransferase and serum albumin of patients between 1 month and 1 year after transplantation. None of the patients developed serious renal or hepatic dysfunction (Table 1).

Comparison and correlation between the dose-adjusted AUC_0–24_ (AUC_0–24_/D) or dose-adjusted C_0_ (C_0_/D) of tacrolimus and each clinical characteristic of the patients, and the AUC_0–12_/D and C_0_/D of everolimus at 1 month and 1 year after transplantation are listed in Table 2. There were significant differences in the AUC_0–24_/D and C_0_/D of tacrolimus among the 3 *CYP3A5* genotypes at both 1 month and 1 year. In addition, there were also significant correlations with aspartate aminotransferase, alanine aminotransferase, and the AUC_0–12_/D or C_0_/D of everolimus 1 month after renal transplantation.

**Figure 1:**
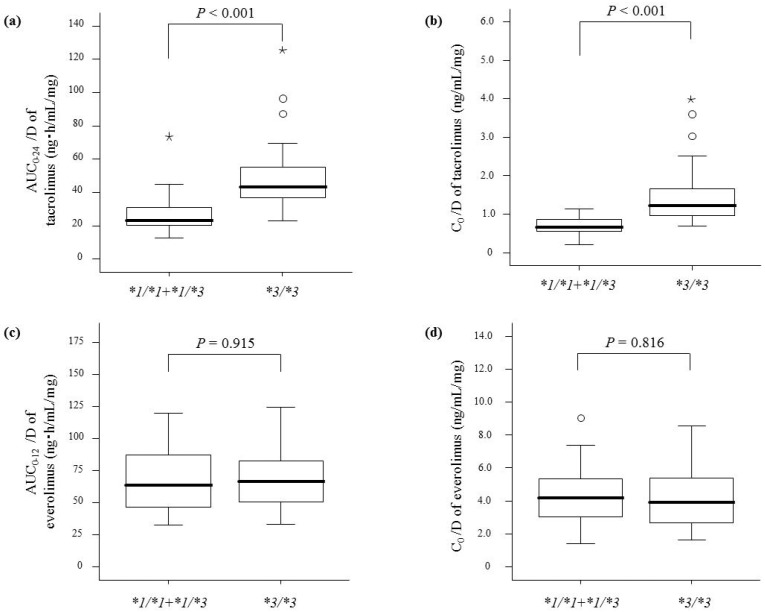
Comparison of dose-adjusted area under the blood concentration-time curves (AUC/D) and trough concentrations (C_0_/D) of tacrolimus and everolimus 1 month after renal transplantation between patients with CYP3A5*1 allele (*n* = 25) and *3/*3 (*n* = 25). Graphical analysis was performed using an SPSS box and whiskers plot. The box spans data between two quartiles (IQR), with the median represented as a bold horizontal line. The ends of the whiskers (vertical lines) represent the smallest and largest values that were not outliers. Outliers (circles) are values between 1.5 and 3 IQRs from the end of the box. Values more than three IQRs from the end of the box are defined as extreme (asterisk). (**a**) AUC_0__–__24_/D of tacrolimus; (**b**) C_0_/D of tacrolimus; (**c**) AUC_0__–__12_/D of everolimus; (**d**) C_0_/D of everolimus.

**Figure 2:**
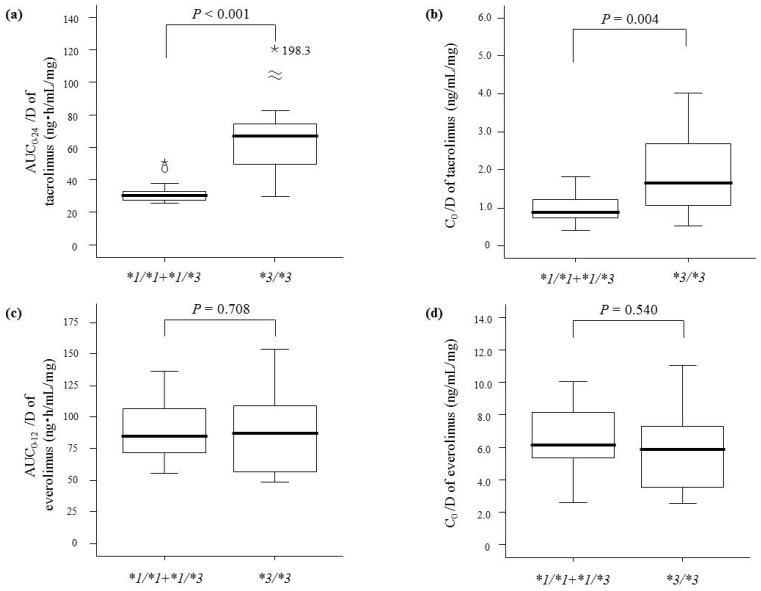
Comparison of the dose-adjusted area under the blood concentration-time curves (AUC/D) and trough concentrations (C_0_/D) of tacrolimus and everolimus 1 year after renal transplantation between patients with CYP3A5*1 allele (*n* = 13) and *3/*3 (*n* = 18). Graphical analysis was performed using an SPSS box and whiskers plot. The box spans data between two quartiles (IQR), with the median represented as a bold horizontal line. The ends of the whiskers (vertical lines) represent the smallest and largest values that were not outliers. Outliers (circles) are values between 1.5 and 3 IQRs from the end of the box. Values more than three IQRs from the end of the box are defined as extreme (asterisk). (**a**) AUC_0__–__24_/D of tacrolimus; (**b**) C_0_/D of tacrolimus; (**c**) AUC_0__–__12_/D of everolimus; (**d**) C_0_/D of everolimus.

Although the AUC_0–24_/D and C_0_/D of tacrolimus were higher for patients with CYP3A5*3/*3 than those with the CYP3A5*1 allele at both 1 month (Figure 1a,b) and 1 year (Figure 2a,b) after transplantation, there were no differences in the corresponding parameters of everolimus at either time point (Figure 1c,d and Figure 2c,d).

**Figure 3:**
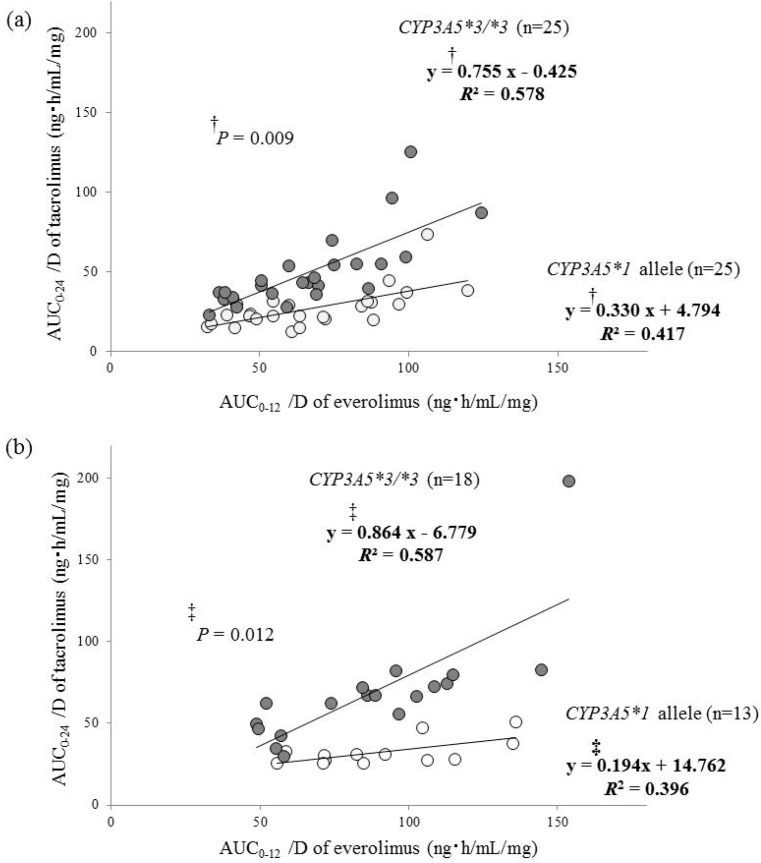
Correlation between the dose-adjusted area under the blood concentration-time curves of tacrolimus and everolimus. (**a**) one month after renal transplantation; (**b**) one year after renal transplantation. Open circles, patients with the CYP3A5*1 allele; closed circles, patients with the CYP3A5*3/*3.

Significant correlations between the AUC_0–24_/D of tacrolimus and AUC_0–12_/D of everolimus were found for patients with the CYP3A5*1 allele or CYP3A5*3/*3 at both 1 month and 1 year after transplantation (Figure 3). The determination coefficients (*R*^2^) were higher for patients with CYP3A5*3/*3 than those with the CYP3A5*1 allele at both 1 month and 1 year after transplantation (0.578 vs. 0.417 and 0.587 vs. 0.396, respectively, Figure 3a,b). In addition, the slopes of regression equations were also larger for patients with CYP3A5*3/*3 than those with the CYP3A5*1 allele at both stages (0.775 vs. 0.330, ^†^ *p* = 0.009 and 0.864 vs. 0.194, ^‡^ *p* = 0.012, respectively, Figure 3a,b).

Stepwise selection multiple linear regression analysis of explanatory variables for the AUC_0–24_/D and C_0_/D of tacrolimus at 1 month and 1 year after transplantation are shown in Table 3. The AUC_0–12_/D and C_0_/D of everolimus and *CYP3A5* genotype (CYP3A5*3/*3) were independent factors influencing the AUC_0–24_/D or C_0_/D of tacrolimus at both stages (all *p* < 0.01). The determination coefficients for the AUC_0–24_/D of tacrolimus at both stages were 0.6 and greater.

**Figure 4:**
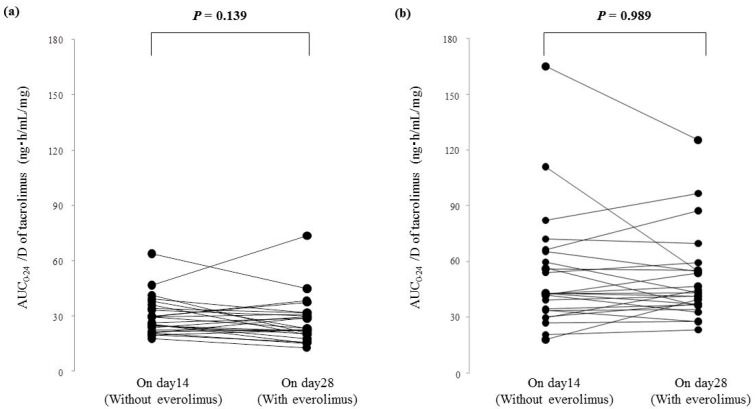
Comparison of the dose-adjusted area under the blood concentration-time curve from 0 to 24 h (AUC_0–24_) of tacrolimus between day 14 and day 28 after renal transplantation. (**a**) Patients with the CYP3A5*1 allele; (**b**) patients with the CYP3A5*3/*3.

There were no changes in the AUC_0–24_/D of tacrolimus from day 14 (without everolimus) to day 28 (with everolimus) for patients with either the CYP3A5*1 allele or CYP3A5*3/*3 (Figure 4a,b).

Correlations with the dose-adjusted maximal plasma concentration (C_max_/D) or the elimination half-life between tacrolimus and everolimus in patients with the CYP3A5*1 allele or CYP3A5*3/*3 at 1 month and 1 year after renal transplantation are listed in Table 4. At both stages, the *r* of the C_max_/D or the elimination half-life were higher for patients with CYP3A5*3/*3 than those with the CYP3A5*1 allele, and the *r* of C_max_/D in each *CYP3A5* genotype was higher than those of the elimination half-life (0.603 > 0.459 for CYP3A5*1 allele and 0.659 > 0.587 for CYP3A5*3/*3 at 1 month; 0.349 > 0.099 and 0.769 > 0.341 at 1 year, respectively).

**Figure 5:**
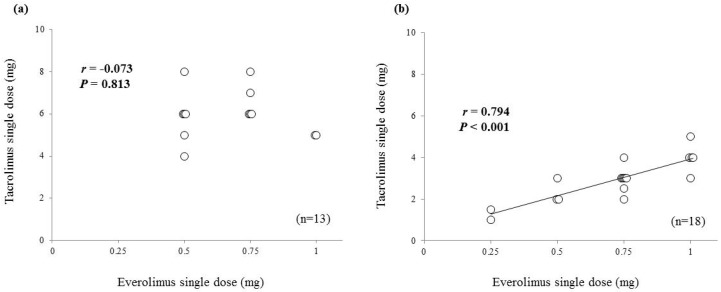
Correlation between the doses of tacrolimus and everolimus 1 year after renal transplantation. (**a**) Patients with the CYP3A5*1 allele; (**b**) patients with the CYP3A5*3/*3.

At 1 year after renal transplantation, there was no correlation between single doses of tacrolimus and everolimus in patients with the CYP3A5*1 allele (Figure 5a, *r* = −0.073, *p* = 0.813); however, a significant correlation was found in patients with the CYP3A5*3/*3 (Figure 5b, *r* = 0.794, *p* < 0.001). 

## 3. Discussion

In the present study at 1 month and 1 year after renal transplantation, 61.6% and 63.3%, respectively, of the variability of the AUC_0–24_/D of tacrolimus were predicted by the combination of *CYP3A5* genotype and AUC_0–12_/D of everolimus. These findings show that the individual AUC of tacrolimus at steady-state can be roughly approximated based on *CYP3A5* activity and the activity of CYP3A4 as a marker of everolimus AUC.

Prior to and after everolimus co-administration, there were no significant differences in the AUC_0–24_/D of tacrolimus for patients with either the CYP3A5*1 allele or CYP3A5*3/*3. We have previously reported that there is no drug interaction between tacrolimus and everolimus affecting pharmacokinetics [15]. In addition, the pharmacokinetics of everolimus are not influenced by *CYP3A5* polymorphisms [11,13,16]. Clinicians may be able to predict the AUC_0–24_/D of tacrolimus based on assessment of CYP3A4 activity by using the AUC_0–24_/D of everolimus, because a drug interaction via CYP3A4/5 does not seem to occur with a combination of these drugs. On the other hand, midazolam is used as the gold standard probe for in vivo CYP3A4 phenotyping [17,18]. De Jonge et al. reported that about 60% of the variability of the AUC_0–24_/D of tacrolimus can be explained by *CYP3A5* genotype and apparent oral clearance of midazolam [19]. In addition, they reported that the pharmacokinetics of tacrolimus in the first year after renal transplantation in patients with the CYP3A5*3/*3 could partly be explained by CYP3A4 activity based on the pharmacokinetics of midazolam and haematocrit [20]. This report supports our results. Similar to tacrolimus, everolimus is extensively bound to red blood cells [10]. Because an affector for red blood cell count is included in blood concentrations of everolimus, hemoglobin might not be an independent factor explaining the variability of the AUC_0–24_/D of tacrolimus in the present study. In patients taking tacrolimus, everolimus may play a role as an indicator of CYP3A4 activity.

The AUC_0–24_/D of tacrolimus was low in patients with a low AUC_0–12_/D of everolimus at 1 month and 1 year after renal transplantation (Figure 3). Therefore, such patients should be carefully monitored to prevent acute rejection. On the other hand, the AUC_0–24_/D of tacrolimus was high in patients with a higher AUC_0–12_/D of everolimus, and this tendency was more pronounced in patients with CYP3A5*3/*3 than those with the CYP3A5*1 allele. In patients with the CYP3A5*1 allele, tacrolimus is metabolized by both CYP3A4 and *CYP3A5*, whereas in patients with CYP3A5*3/*3, tacrolimus is solely metabolized by CYP3A4. Therefore, patients with a higher AUC_0–12_/D of everolimus and the CYP3A5*3/*3 need to be especially carefully monitored for tacrolimus-induced side effects. Shihab et al. have reported that an everolimus trough concentration of 3.8 ng/mL is needed to preserve a balance of efficacy and safety in the maintenance phase after renal transplantation for patients taking a low dose of tacrolimus [9]. Interestingly, in the present study, the correlation between the daily doses of tacrolimus and everolimus at 1 year after renal transplantation was extremely high in patients with CYP3A5*3/*3 (Figure 3b). Since clinicians adjust the daily dose of tacrolimus and everolimus based on their blood concentrations considering the efficacy and safety for immunosuppressive drugs through 1 year after transplantation, a good correlative relationship between the daily doses of tacrolimus and everolimus might be observed.

At 1 month and 1 year after renal transplantation, the correlation coefficients between the C_max_/D or the elimination half-life of tacrolimus and everolimus were higher for patients with CYP3A5*3/*3 than those with the CYP3A5*1 allele, and the correlation coefficients of C_max_/D were higher than those of elimination half-life for each *CYP3A5* genotype. In addition, we have reported that the larger inter-individual variability of tacrolimus bioavailability for oral formulations is influenced by *CYP3A5* polymorphism [21]. These results suggest that the inter-individual variability of tacrolimus pharmacokinetics in patients without *CYP3A5* activity depends strongly on CYP3A4 activity in the small intestine. On the other hand, significant differences in body weight, aspartate aminotransferase and serum albumin of recipients between 1 month and 1 year after renal transplantation were found. However, in our previous study [22], the AUC_0–24_/D and C_0_/D of tacrolimus were unaffected by these factors. Therefore, these differences in each stage do not affect the interpretation of the results in this study.

Tacrolimus and everolimus are substrates of the drug transporter *P*-glycoprotein [1,2,10]. In the present study, we did not assess the activity of *P*-glycoprotein; however, our results may indicate an influence of *P*-glycoprotein, although it is unclear how much pharmacokinetic variability of either drug is related to *P*-glycoprotein. Vanhove et al. reported that the pharmacokinetic parameters of fexofenadine, a substrate of *P*-glycoprotein, were not predictive of tacrolimus oral clearance [23]. On the other hand, there is not enough evidence that *ABCB1* polymorphisms are useful as a factor in dose adjustment for tacrolimus [24,25] or everolimus [11,26]. Therefore, further studies addressing this topic might be necessary. However, as shown in Figure 5, a good correlation between the single dose of tacrolimus and everolimus was found in patients with CYP3A5*3/*3 in the maintenance phase after renal transplantation. As to our protocol, when everolimus is added to the regimen of patients taking tacrolimus, clinicians may be able to individualize the initial dose of everolimus based on the AUC_0–24_/D or C_0_/D of tacrolimus and knowledge of the *CYP3A5* genotype. However, further study of this approach is necessary. 

Our results could be interpreted within the context of the study limitations. The present study was retrospectively performed with a small patient group in a non-controlled single-center study. In addition, the effect of pharmacokinetics of tacrolimus or everolimus on clinical outcome was not analyzed. Hence, additional studies with larger sample sizes might be necessary. 

In conclusion, the variability of the AUC_0–24_/D of tacrolimus for each *CYP3A5* genotype could be predicted based on the AUC_0–12_/D of everolimus. This finding seems to show that everolimus AUC might reflect an impact of CYP3A4 activity on tacrolimus AUC. Indicators of the *CYP3A5* genotype and everolimus AUC may be especially useful for evaluating the metabolic activity of CYP3A4/5 for tacrolimus in the small intestine. For renal transplant recipients, clinicians may be able to comprehensively carry out dose adjustments of tacrolimus and everolimus based on the relationship of AUCs of both drugs in each *CYP3A5* genotype.

## 4. Materials and Methods

### 4.1. Patients and Protocols

This retrospective study enrolled 50 Japanese renal transplant recipients who received renal grafts between October 2013 and March 2017 and were administered tacrolimus (modified-release once-daily formulation (Graceptor^®^, Astellas, Tokyo, Japan)) and everolimus (Certican^®^, Novartis Pharma, Tokyo, Japan). The study protocol was approved by the Ethics Committee of Akita University School of Medicine (Protocol No. 1248, 25 November 2014), and all patients gave written informed consent. The patient eligibility criteria for the study were (1) a tacrolimus-based immunosuppressive regimen including mycophenolate mofetil (MMF; Cellcept^®^, Chugai Pharmaceutical, Tokyo, Japan), basiliximab, and steroid; (2) an absence of pre-transplant donor-specific antibodies or delayed graft function; (3) no severe liver dysfunction or gastrointestinal motility; and (4) no introduction of drugs or foods that obviously affect CYP3A function during the study period. All patients received rabeprazole and the sulfamethoxazole-trimethoprin drug combination. Patients initially received a combination immunosuppressive therapy regimen of tacrolimus and MMF 2 days prior to renal transplantation. An initial oral dose (0.20 mg/kg) of tacrolimus was given every 24 h at a designated time (09:00). Patients received a 24 h continuous intravenous infusion (CIV) of tacrolimus (0.05 mg/kg/day) beginning on the day of transplantation until day 3. On day 3, tacrolimus administration was changed from CIV to oral administration. An initial oral dose of MMF 1500 mg/day was given in equally divided doses every 12 h at designated times (09:00 and 21:00). On day 14, immediately after blood collection for tacrolimus analysis, everolimus was added to the above combination therapy at an initial dose of 1.5 mg/day in equally divided doses every 12 h at designated times (09:00 and 21:00). The target C_0_ of tacrolimus was 15–20 ng/mL during CIV, 10–12 ng/mL during the first week, 8–10 ng/mL during the second to fourth week after renal transplantation, and 5–8 ng/mL thereafter. The target C_0_ of everolimus was 3–5 ng/mL after the second week. All patients received a controlled hospital diet served daily at 7:30, 12:30, and 18:00 in the hospital for the pharmacokinetic study.

### 4.2. Sample Collection and Analytical Methods

After renal transplantation, serial whole blood samples were collected in EDTA-2Na tubes just prior to the morning doses of tacrolimus and everolimus. An initial TDM of everolimus was carried out the second week after the start of administration. Blood samples for tacrolimus and everolimus analysis were collected just prior to and 1, 2, 3, 4, 6, 12, and 24 h after the morning doses (blood collection 24 h after drug administration was carried out only for tacrolimus) on day 14 (blood collection on day 14 was carried out only for tacrolimus), on day 28 (at 1 month), and at 1 year. Blood samples of tacrolimus and everolimus were collected just prior to the morning doses every month during the follow-up period. Blood concentrations of tacrolimus were determined by the chemiluminescence magnetic microparticle immunoassay (CLIA) on the Architect-i1000^®^ system (Abbott Laboratories; Abbott Park, IL, USA) according to the manufacturer’s instructions. Blood concentrations of everolimus were determined by the latex agglutination turbidimetric immunoassay (LTIA) on the CA-90^®^ chemistry analyzer (Furuno Electric Company, Nishinomiya, Japan) according to the manufacturer’s instructions. 

### 4.3. Genotyping

DNA was extracted from whole blood samples before renal transplantation in 50 recipients with a QIAamp Blood Kit (Qiagen, Hilden, Germany) and was stored at −80 °C until being analyzed. For genotyping the *CYP3A5* 6986A > G (*3), the polymerase chain reaction-restriction fragment length polymorphism (PCR-RFLP) method was used. PCR was performed with a 20 µL aliquot containing 50 ng of genomic DNA, 50 pmol of each primer, 80 µM of each deoxynucleotide triphosphate, 0.6 units of Ampli-Taq Gold DNA polymerase, 1.2 mM MgCl_2_, and 1× reaction buffer (Applied Biosystems, Foster City, CA, USA). The primers used were as follows: forward, 5′-ATGGAGAGTGGCATAGGAGATA-3′; reverse, 5′-TGTGGTCCAAACAGGGAAGAAATA-3′. PCR amplification conditions were 8 min of initial denaturation at 94 °C, followed by 40 cycles of melting at 94 °C for 30 s, annealing at 55 °C for 30 s, and elongation at 72 °C for 30 s, followed by a final elongation for 10 min at 72 °C. The PCR products were digested at 37 °C overnight with 10 units of *SspI* (New England Biolabs, Inc., Beverly, MA, USA). Digested products were separated on 2.5% agarose gel containing ethidium bromide. When the A allele (CYP3A5*1 allele) was present, the 130 bp PCR fragment was divided into 107 bp and 23 bp fragments [27]. The analysis results obtained from PCR-RFLP were confirmed with a fully automated single nucleotide polymorphism (SNP) detection system (prototype i-densy^®^, Arkray Inc., Kyoto, Japan).

### 4.4. Pharmacokinetic Analysis

Pharmacokinetic analyses of tacrolimus and everolimus were carried out using the standard non-compartmental method with Phoenix WinNonlin Version 6.4 (Pharsight Co., Mountain View, CA, USA). The AUC from 0 to 12 h (for everolimus) and 0 to 24 h (for tacrolimus) were calculated using the linear trapezoidal rule. The C_max_ of tacrolimus was obtained directly from the profile. The elimination half-life of tacrolimus was obtained using log-linear regression of the terminal phase of the concentration-time data with at least three sampling points.

### 4.5. Statistical Procedures

The Shapiro–Wilk test was used to assess distribution. The characteristics of renal transplant recipients and parameters for tacrolimus and everolimus were expressed as medians (quartile 1–quartile 3). The Kruskal–Wallis test or Mann–Whitney U test was used to determine the difference in continuous values between groups. The chi-square test was used to examine differences in categorical data, except when the expected number of cells was <5, in which case Fisher’s exact test was used. The analysis of covariance was used to examine the parallelism of slopes between two regression equations. The Wilcoxon signed-rank test was used to determine the inter-patient difference in continuous values. Spearman’s rank correlation coefficient test was used to assess correlations in continuous values between groups, and all results were expressed as a correlation coefficient of determinant (*r*). Stepwise multiple linear regression analysis was performed to determine the effect of all factors in a univariate analysis. For each patient, the *CYP3A5* genotype was replaced with dummy variables (1 and 0, 0 and 1, and 0 and 0, respectively). The percent variation that could be explained by the multiple regression equation was expressed as a coefficient of determination (*R*^2^). A *p*-value less than 0.05 was considered statistically significant. Statistical analysis was performed with SPSS 20.0 for Windows (SPSS IBM Japan Inc., Tokyo, Japan).

## Figures and Tables

**Table 1:** Clinical characteristics of patients after renal transplantation.

Items	1 Month after Transplantation		1 Year after Transplantation	
Gender				
Male	30	(60.0%)	20	(64.5%)
Female	20	(40.0%)	11	(35.5%)
Tacrolimus single dose (mg)	8.0	(5.0–12.0)	4.0	(3.0–6.0)
Tacrolimus C_0_ (ng/mL)	7.0	(5.9–8.5)	4.5	(4.0–6.0)
Everolimus single dose (mg)	0.75		0.75	(0.50–0.75)
Everolimus C_0_ (ng/mL)	3.2	(2.4–4.1)	3.5	(3.0–4.5)
Age (year)	55.0	(47.0–61.0)	58.0	(52.5–62.5)
Body weight (kg)	56.3	(47.2–63.3)	59.9	(53.9–66.0)
Aspartate aminotransferase (IU/L)	14	(11–17)	22	(17–25)
Alanine aminotransferase (IU/L)	14	(9–21)	16	(12–24)
Hemoglobin (g/dL)	10.4	(9.6–11.4)	11.9	(10.8–13.3)
Serum albumin (g/dL)	3.8	(3.5–4.0)	4.1	(3.8–4.4)
Creatinine clearance (mL/min)	50.3	(41.3–60.3)	54.2	(39.4–63.9)
*CYP3A5* genotype				
*1/*1	5	(10.0%)	3	(9.7%)
*1/*3	20	(40.0%)	10	(32.2%)
*3/*3	25	(50.0%)	18	(58.1%)

The values are expressed as median (quartile 1–quartile 3) or number (%) of patients. C_0_, trough concentration.

**Table 2:** Comparison and correlation with the dose-adjusted AUC_0–24_ and C_0_ of tacrolimus and clinical characteristics of recipients.

Tacrolimus	1 Month after Transplantation				1 Year after Transplantation			
	AUC_0–24_/D (ng·h/mL/mg)	*p*-Value	C_0_/D (ng/mL/mg)	*p*-Value	AUC_0–24_/D (ng·h/mL/mg)	*p*-Value	C_0_/D (ng/mL/mg)	*p*-Value
Gender		0.428		0.663		0.113		0.261
Male	35.8 (23.1–46.6)		0.89 (0.71–1.36)		59.0 (30.4–73.7)		1.42 (0.90–2.46)	
Female	32.2 (22.8–39.2)		0.86 (0.63–1.17)		34.9 (30.6–48.1)		1.03 (0.93–1.43)	
*CYP3A5* genotype		<0.001		<0.001		<0.001		0.019
*1/*1	20.3 (15.7–22.3)		0.58 (0.42–0.59)		27.5 (26.6–29.2)		0.80 (0.59–1.15)	
*1/*3	26.0 (21.3–31.3)		0.72 (0.57–0.87)		30.6 (27.3–37.7)		0.93 (0.72–1.20)	
*3/*3	43.1 (36.7–54.8)		1.20 (0.97–1.66)		66.6 (49.6–74.5)		1.63 (1.03–2.67)	
	Correlation coefficient (*r*)	*p*-Value	Correlation coefficient (*r*)	*p*-Value	Correlation coefficient (*r*)	*p*-Value	Correlation coefficient (*r*)	*p*-Value
Age (year)	0.219	0.127	0.236	0.100	0.345	0.058	0.307	0.092
Body weight (kg)	0.021	0.883	−0.018	0.903	0.038	0.839	0.122	0.515
Aspartate aminotransferase (IU/L)	0.346	0.014	0.345	0.014	0.167	0.370	−0.139	0.457
Alanine aminotransferase (IU/L)	0.436	0.002	0.392	0.005	0.150	0.421	−0.019	0.919
Hemoglobin (g/dL)	0.145	0.315	0.274	0.054	−0.024	0.900	0.008	0.965
Serum albumin (g/dL)	−0.014	0.921	0.073	0.615	−0.156	0.401	−0.141	0.448
Creatinine clearance (mL/min)	0.116	0.423	0.115	0.426	−0.259	0.160	−0.054	0.772
Everolimus pharmacokinetics	0.527 *	<0.001	0.526 **	<0.001	0.442 *	0.013	0.258 **	0.161

The values are expressed as median (quartile 1–quartile 3) or correlation coefficient. AUC_0–24_, area under the concentration–time curve from 0 to 24 h; C_0_, trough concentration; D, tacrolimus single dose; * vs. dose adjusted AUC_0–12_ of everolimus (ng·h/mL/mg); ** vs. dose-adjusted C_0_ of everolimus (ng/mL/mg).

**Table 3:** Stepwise multiple regression analysis of explanatory variables for the dose-adjusted AUC_0–24_ and C_0_ of tacrolimus.

Objective Variable	Explanatory Variable	Slope	SE	SRC	*p*-Value	*R*^2^
AUC_0–24_/D of tacrolimus at 1 month after transplantation (ng·h/mL/mg)						0.616
	AUC_0–12_/D of everolimus (ng·h/mL/mg)	0.534	0.083	0.581	<0.001	
	*CYP3A5* genotype (*3/*3 = 1)	23.360	3.917	0.539	<0.001	
	Intercept =	−9.038	6.27			
C_0_/D of tacrolimus at 1 month after transplantation (ng/mL/mg)						0.643
	C_0_/D of everolimus (ng/mL/mg)	0.246	0.034	0.627	<0.001	
	*CYP3A5* genotype (*3/*3 = 1)	0.792	0.135	0.510	<0.001	
	Intercept =	−0.379	0.181			
AUC_0–24_/D of tacrolimus at 1 year after transplantation (ng·h/mL/mg)						0.633
	*CYP3A5* genotype (*3/*3 = 1)	38.899	7.563	0.590	<0.001	
	AUC_0–12_/D of everolimus (ng·h/mL/mg)	0.641	0.130	0.568	<0.001	
	Intercept =	−26.058	13.137			
C_0_/D of tacrolimus at 1 year after transplantation (ng/mL/mg)						0.427
	*CYP3A5* genotype (*3/*3 = 1)	0.943	0.252	0.539	0.001	
	C_0_/D of everolimus (ng/mL/mg)	0.160	0.053	0.435	0.005	
	Intercept =	−0.025	0.387			

AUC_0–24,_ and AUC_0–12,_ area under the concentration–time curve from 0 to 24 h, and 0 to 12 h; C_0_, trough concentration; D, tacrolimus or everolimus single dose. SE, standard error; SRC, standardized regression coefficient.

**Table 4:** Correlation between the dose-adjusted C_max_ and elimination half-life of tacrolimus and everolimus in each *CYP3A5* genotype at 1 month and 1 year after renal transplantation.

*CYP3A5* Genotype	C_max_/D		Elimination Half-Life	
	Correlation Coefficient (*r*)	*p*-Value	Correlation Coefficient (*r*)	*p*-Value
1 month after transplantation				
CYP3A5*1allele	0.603	<0.001	0.459	0.021
*3/*3	0.659	<0.001	0.587	0.002
1 year after transplantation				
CYP3A5*1allele	0.349	0.243	0.099	0.748
*3/*3	0.769	<0.001	0.341	0.181

C_max_, maximum blood concentration; D, tacrolimus single dose; *r*, tacrolimus vs. everolimus in C_max_ or half-life.
